# Case report: Diagnosis and autogenous vaccine treatment of herpesvirus in a green turtle (*Chelonia mydas*) in Santa Marta, Colombia

**DOI:** 10.3389/fvets.2024.1258209

**Published:** 2024-01-31

**Authors:** Lyda R. Castro, Vivian Villalba-Viscaíno, Ángel Oviedo, Edgar Zambrano, Angela Dávila, Gualberto Naranjo, Blanca De Oro-Genes, Anthony Combatt, Julieth Prieto-Rodríguez, Arnaldo Ortiz, Natalia Villamizar

**Affiliations:** ^1^Centro de Genética y Biología Molecular. Grupo de Investigación Evolución, Sistemática y Ecología molecular, Universidad del Magdalena, Santa Marta, Colombia; ^2^Grupo de Investigación en Inmunología y Patología, Universidad del Magdalena, Santa Marta, Colombia; ^3^Centro de Vida Marina, Santa Marta, Colombia; ^4^Corporación Autónoma Regional del Magdalena (CORPAMAG), Santa Marta, Colombia

**Keywords:** chelonid herpesvirus 5, PCR, DNA polymerase gene, fibropapilloma, tumor recovery

## Abstract

This study reports the first case of fibropapillomatosis (FP) in the green turtle *Chelonia mydas* that has been successfully diagnosed and treated in Colombia. Worldwide, FP has reached epizootic proportions as it has been reported in marine turtles of tropical and subtropical waters, and in severe cases, it reduces the probability of survival. Treatment has been elusive as multiple surgical excisions are needed due to tumor recurrence. In this case, one green turtle with multiple tumors was diagnosed by histopathology and molecular detection of the chelonid herpesvirus 5 (ChHV5) by means of amplification and sequencing of the DNA polymerase (DNApol) gene. Two separate treatments that consisted of autogenous vaccines and surgical excisions were applied; the first one had a partial success as one out of the tumors treated reappeared after 3 months post-treatment. Treatment 2 consisted of an autogenous vaccine enriched with adjuvants and applied at increasing doses, after which, the tumor significatively decreased in size and was surgically removed. At the end of the 6 months follow-up period, no tumor recurrence was observed, and the turtle was in apparent optimal health conditions. These findings, although limited, suggest a possible treatment that might help to contain this epizootic problem.

## 1 Introduction

The green turtle *Chelonia mydas*, one of the four marine turtle species that visit the coasts of the Colombian Caribbean, is classified as endangered (EN) according to the International Union for Conservation of Nature (IUCN) red list of threatened species^1^ and the Red Book of Reptiles of Colombia, mainly due to the deterioration of its habitat, illegal hunting, destruction of nesting areas and diseases (1, 2). Since the last century, a debilitating neoplastic disease known as fibropapillomatosis (FP), has been reported globally in marine turtles of tropical and subtropical waters (3–6). FP causes, among others, external and internal tumors, anaemia, anorexia, acidosisis and mortalities linked to immunosuppression, secondary infections, and opportunistic pathogens (7–10).

The presence of FP has been associated with poor coastal environmental conditions (eutrophication, nitrogen-footprints, invasive macroalgae) and species-specific factors (age, size, foraging ecology) (3, 11). Also, FP has been mostly associated with a herpesvirus, which is currently considered the most likely aetiological agent of this disease (5, 12, 13). Herpesviruses are members of the family Herpesviridae, a large taxon of DNA viruses that have been described in most vertebrate animals, including reptiles (14). Reptilian herpesviruses are now classified in the subfamily Alphaherpesvirinae, and the virus found in Testudines has been classified in the genus *Scutavirus* (9). A specific herpesvirus called chelonid herpesvirus 5 (ChHV5) has been isolated from the lesions present in turtles (12, 15). More recently, however, both ChHV5 and *C. mydas* papillomavirus 1 (CmPV1) DNA have been detected in FT tumor tissues, indicating that both viruses may be at play in this disease (16, 17).

The distribution of the disease is wide, being identified in different species of sea turtles throughout the world, which is why it has been considered to be of epizootic proportions. The spread of the disease has mainly affected the green turtles *C. mydas* that inhabit waters in equatorial and sub-equatorial regions (18). Some authors have reported that the global prevalence of FP ranges between 0 to 92%, having an average prevalence between 15.41% to 42.11% between the years 2010 to 2013 (19). More recent studies document important figures of disease prevalence of 13.2% to 35.3% in the northeastern region of Brazil (18), and of 50% in Florida (20). In Colombia we are not aware of reports of FP prevalence (21).

Despite the incidence of the disease, the pathophysiological mechanisms involved in the disease, and the causes of its progression and recurrences, which are very frequent, are not known (18). In general, information regarding the pathophysiological mechanisms related to FP and herpesvirus infections is scarce. Additionally, current treatment options of reptilian HVs are very limited, with only one case of an autogenous vaccine treatment reported in turtles (22). This study presents the case and treatment of a green turtle, *C. mydas*, diagnosed with fibropapillomatosis and treated with an autogenous vaccine, followed by a 6 months observation period.

## 2 Case description

### 2.1 Clinical signs and diagnosis

A juvenile green turtle *C. mydas*, rescued by the Center for Attention, Assessment and Rehabilitation of Marine Fauna—CAV-R marine of the Regional Autonomous Corporation of Magdalena—CORPAMAG, and Center of Marine Life, was received on November 08, 2020, delivered by fishermen of Tasajera, Magdalena (10.9784181, −74.3264348). The individual was received with multiple papillomatous lesions located on the right rear flipper, right eye region, and neck. The turtle weighed 20 kg, with CCL: 54 cm and CCW: 48 cm.

Macroscopically, the lesions corresponded to gray, pink, and brown masses of firm consistency with multiple exophytic papillary growths. Representative sections of 2–3 mm were formalin fixed, trimmed, embedded in paraffin, cut in 2–4 μm sections and stained with hematoxylin and eosin (H&E) according to standardized procedures.

**Figure 1:**
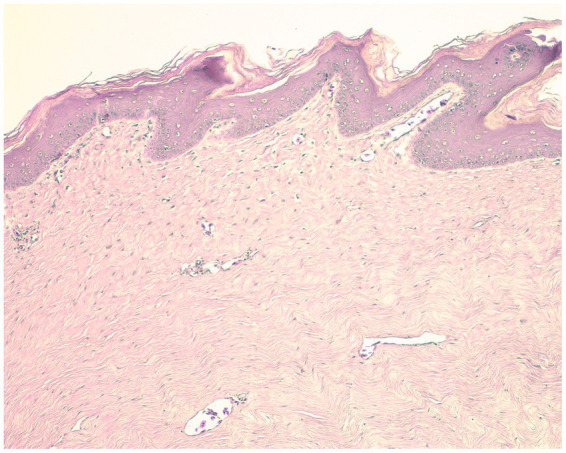
Histological sections. Hematoxylin and eosin stain. 10×.

Histological sections showed a benign neoplasm with an epithelial and mesenchymal component. The epidermis presented moderate hyperkeratosis and epidermal hyperplasia with papillomatosis. In the dermis there was proliferation of dermal fibroblasts, without cellular atypia, mild angiogenesis, and scant inflammatory infiltrate, predominantly lymphocytic (Figure 1). The proliferative changes observed in the analyzed tissue indicated that the described histopathological findings were consistent with previous descriptions of fibropapilloma (FP) lesions produced by potential different types of herpesvirus infections (23).

To carry out the molecular diagnosis, a blood sample was taken by puncture of the dorsal cervical sinus of the turtle, positioning the animal at 45 degrees, with the head lower than the rest of the body (24). After asepsis and antisepsis techniques with sterile iodized solution and 70% alcohol, a total of 3 mL of blood in vacutainer tubes with EDTA anticoagulant was collected (25). Additionally, a sample of the neck lesion was taken after asepsis and antisepsis with iodine solution, followed by 70% alcohol and application of 2% lidocaine anesthetic solution. Partial sections of the tumor were deposited in two microcentrifuge tubes, one containing phosphate buffered saline (PBS) and one with 100% ethanol.

The DNA extraction was carried out from the samples of the tumor tissue and blood samples at the Center of Genetics and Molecular Biology from Universidad del Magdalena. The Lucigen MasterPureTM Complete DNA & RNA purification kit was used for blood and the Omega BIO-TEK E.Z.N.A Tissue DNA kit for tissue, following the manufacturer’s instructions. Detection of Chelonid herpesvirus 5 (ChHV5) was performed by conventional PCR and real-time PCR using specific primers and probe for the DNA polymerase (ADNpol) gene of the ChHV5 virus (17, 26). Conventional PCR and real-time PCR were performed following previously described conditions (17, 27). A sample with nuclease-free water was used as a negative control. The PCR was purified and sequenced in both directions and sequences were submitted to Blast^2^ analyses on NCBI (National Center of Biotechnology Information).

Molecular diagnosis confirmed the presence of the virus both in the tumor tissue sample and in blood using conventional PCR. The qPCR only detected the virus in the tumor tissue sample. Blast analyses of the obtained sequence indicated 100% similarity with other strains of Chelonid herpesvirus 5 (ChHV5) sequences (Sequence IDs: MH144348, MH101744, JN580279, HQ000007). The sequence was submitted to GenBank (Accession No. OR259410).

Furthermore, a phylogenetic analysis was performed with a data matrix including sequences of different ChHV5 strains downloaded from GenBank. The data were analyzed using Geneious prime (28). The alignment by codons was performed using the Mafft algorithm (29) and the matrix was curated with translator X. IQTREE web server (30) was used to find the best substitution model, applying the Bayesian inference criterion (BIC). Also, to perform a Maximum Likelihood analysis using the Bootstrap algorithm with a fast search, applying 10,000 pseudoreplicates.

**Figure 2:**
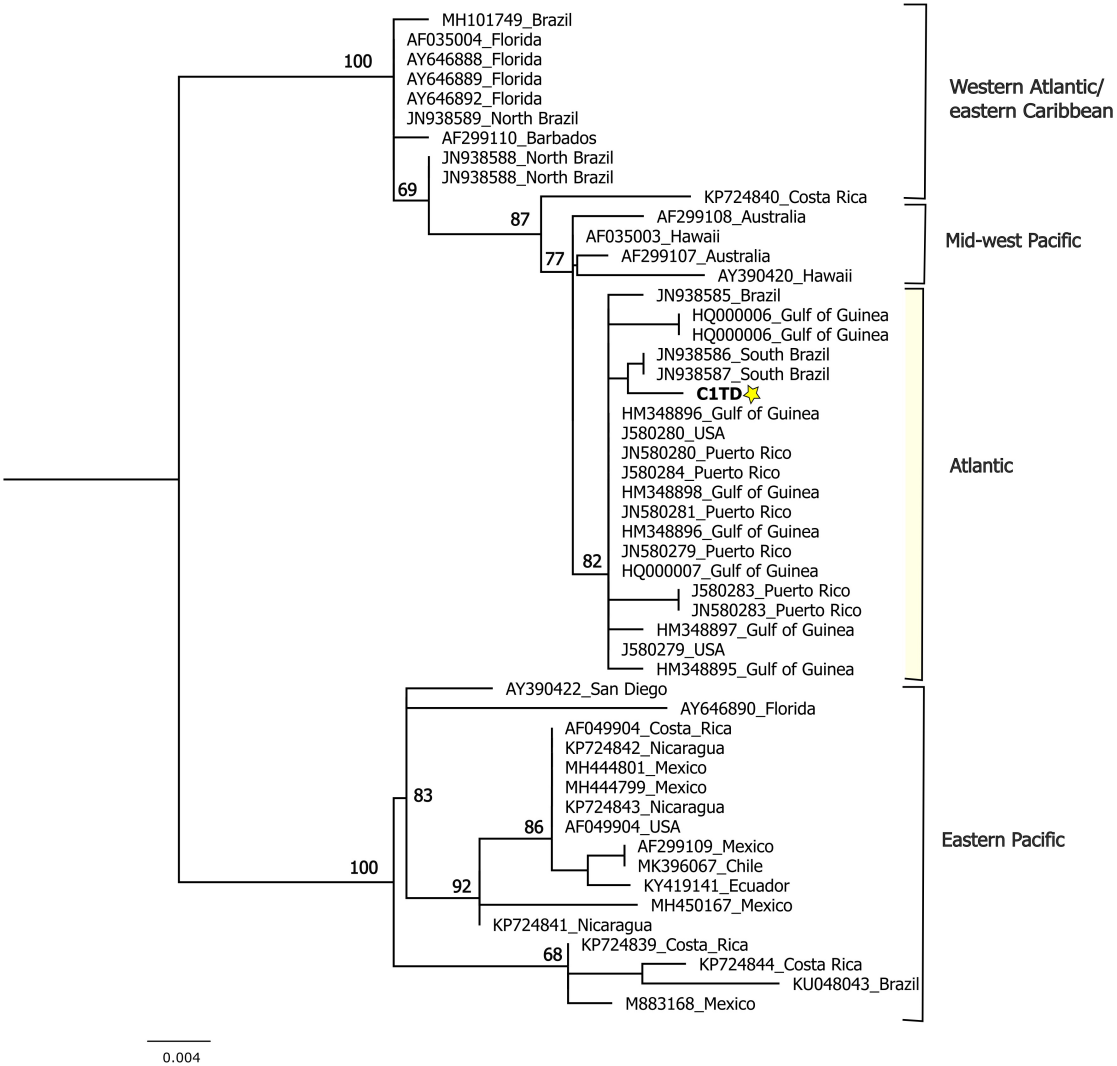
Phylogenetic reconstruction using maximum likelihood including the DNApol sequence obtained in this study and ChHV5 sequences of other strains downloaded from GenBank.

The phylogenetic analysis recovered four phylogeographic groups: Atlantic, West Atlantic-East Caribbean, East Pacific and West-Middle Pacific, previously described in other works (6, 31). The sequence of ChHV5 obtained in this study was closely related to sequences of the Atlantic phylogeographic group comprising Brazil, Puerto Rico and the Gulf of Guinea (Figure 2).

### 2.2 Treatment 1: autogenous vaccine on increasing and decreasing doses

Two cycles of autovaccine were applied. The autogenous vaccine 1 consisted of a macerate of the biopsy (first cycle of treatment), and a macerate of the biopsy mixed with a blood sample from the same individual (second cycle of treatment). These samples were obtained following the procedures described above and formalin was added to both samples. The vaccine was administered in 7 treatments per cycle, through intramuscular puncture, from March 29 to April 2 and from May 1 to May 7 of the year 2021, as shown in Supplementary Table S1.

At the end of the first cycle of the autogenous vaccine 1, no obvious response was observed in relation to the presence or size of the masses. However, at the end of the second cycle, a significant regression was observed, followed up by a surgical remotion of the tumors in May 2021. Although the right rear flipper and right eye tumor area healed completely, there was a recurrence of the neck lesion, being noticeable in August 2021.

### 2.3 Treatment 2: autogenous vaccine (with adjuvants) in increasing doses

A new autogenous vaccine was prepared with tissue excised from the neck lesion. This tissue was ground in liquid nitrogen and then treated with formalin to extract proteins keeping their antigenicity (22). After this pre-treatment, the preparation was centrifuged and sterilized under UV light, and aluminum salts were added as an adjuvant agent. The vaccine was store at −20°C until application. The autovaccine was administered intramuscularly, following a scheme of increased doses, as shown in Supplementary Table S1.

**Figure 3:**
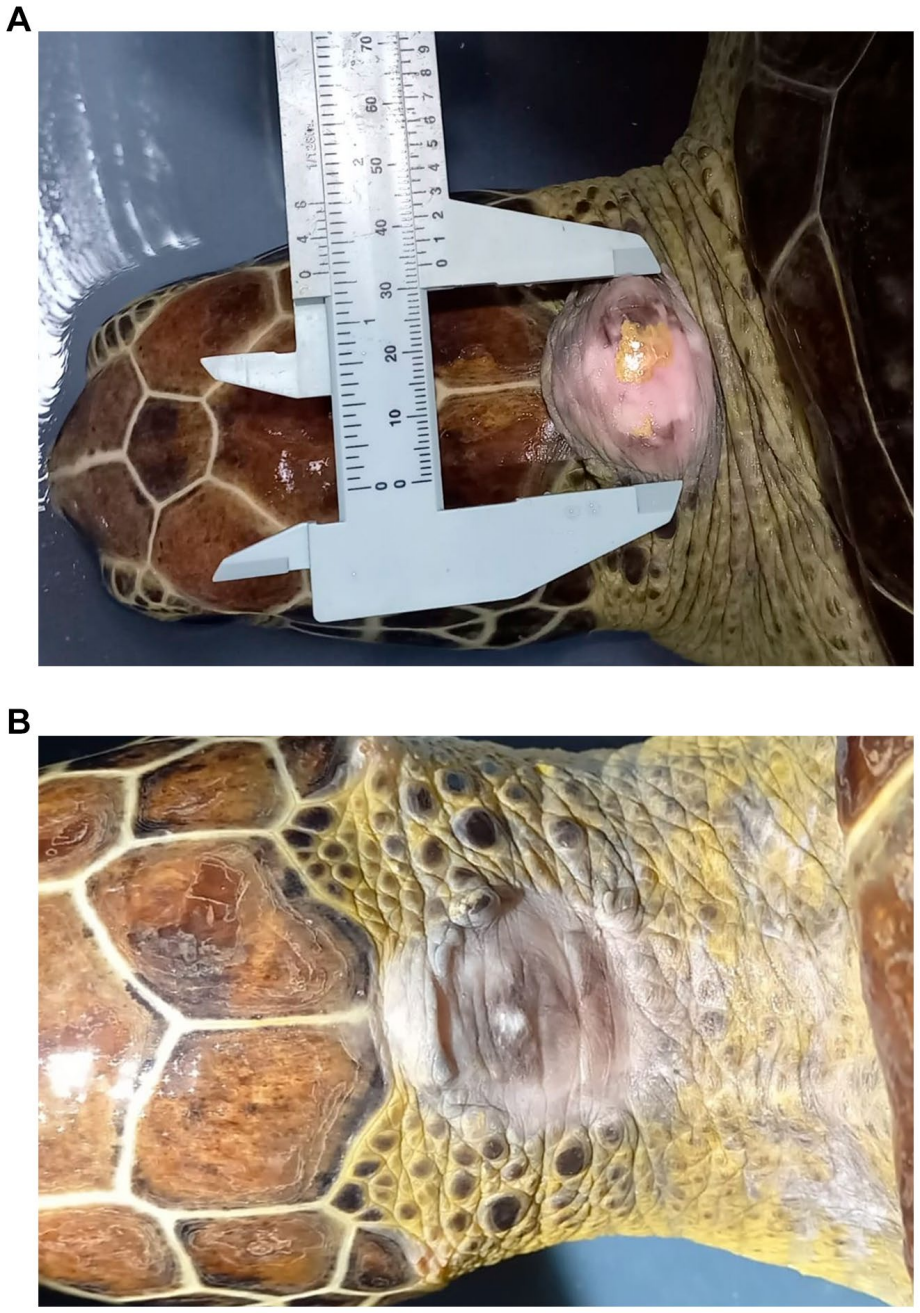
Morphometric and morphological status of the fibropapilloma (FP) at the start of treatment with autovaccine 2 **(A)**. Complete healing without recurrence of the lesion at 6 months post-surgery **(B)**.

The turtle’s health was monitored and verified prior and during the application of the autovaccine 2 doses. After the application of the sixth treatment (September 14, 2022), changes in the size of the FP were recorded, registering a size of 30 × 24.5 mm (Figure 3A). At the end of the proposed scheme, in the month of October 2022, a progression towards the reduction of the tumor size was observed, which for this date was registered with dimensions of 29 × 16 mm. Additionally, a change in the texture and consistency of the FP was also observed, becoming very soft.

Considering the positive response to the autogenous vaccine 2 treatment, and due to the excellent health conditions of the turtle, excision of the tumor remnant was performed in a surgical procedure on November 25, 2022 at the San Francisco Veterinary Hospital in the city of Santa Marta. After the excision, the turtle had a favorable and positive recovery with adequate wound healing according to the expected times. After 6 months of follow up there was no recurrence of the FP, neither in the neck nor in any other regions of the body (Figure 3B).

## 3 Discussion and conclusion

Autogenous vaccine treatments for papilloma viruses have been reported in different animals, especially cattle (32, 33). These studies have shown that autovaccination may provide a therapeutic effect by activating humoral and cellular immunity (32, 33). Recently, an autogenous vaccine therapy was proposed and used for the treatment of HV-associated papillomatosis in Williams’ mud turtle (*Pelusios williamsi*) (22). In their study, the autogenous vaccine induced substantial areas of necrosis of the papillomatous lesions, thus indicating the efficacy of the vaccine (22). To our knowledge, there are no publications on autogenous vaccine therapies for green turtles, *C. mydas*, even though its populations have been highly affected by this disease in the past years (9, 11). One of the limitations in using this type of vaccines in turtles could be the lack of sufficient tumors (in patients) needed to produce adequate vaccine doses (10). Also, the difficulty of conducting experimental research in marine turtles, given their state of conservation and protection (34). One of the advantages, on the other hand, is that the vaccine can be developed in-house at an affordable cost and does not require specialist equipment, which makes it a good option for implementation in countries such as Colombia.

Adjuvants are commonly added to vaccines to elicit maximal immune response because they increase the capacity of antigen-presenting cells to induce T-cell activation and cytokine production, which provide maximum protection against specific pathogens (35). In our study, we added adjuvants to improve the efficacy of the newly developed vaccine. Despite the administration of these types of vaccines could result, as in this case, in a successful recovery, it has been recorded that the effectivity of treatments based on autogenous vaccines depend on different factors, that can be internal, such as: immunity levels, age, or other secondary diseases present in the animal; or external, such as climate, management given to the animal, or alimentation (33). In this sense, it is important to emphasize that ours is a one patient case study and the treatment must be tested case by case, as it might not be effective in other animals. Also, further studies are required to confirm the effects of the autogenous vaccine treatment on the generation of immunity and its true effect on the regression of the tumors, as spontaneous regressions have been reported. However, those records are based on observations of individuals in the wild in which the regressions took a significant amount of time, in some cases, years (36, 37). In the case of marine turtles kept in captivity and the safe release of healthy animals to the wild, effective, and fast treatments are crucial, as well as the clinical follow up of the viral load.

It has been reported that all previously HV infected animals should be treated as latent carriers and potential shedders to naive populations (38). However, according to Reséndiz et al. (39), if the turtle is clinically stable and is capable of fulfilling its vital functions autonomously, even in the presence of FP lesions, this does not represent a limitation for its life, nor does it represent a risk for its own species or other species, so this should not prevent their release into the natural environment. Also, Page-Karjian et al. (40) recommend that turtles that have been treated for this pathology to be released as quickly as possible, since the stress of captivity could contribute to tumor recurrence or the development of new tumors. In that sense, diagnostic turtles with few or no lesions, once treated with the vaccine, should be considered as candidates for liberation.

The most common treatment for FP is surgical removal of the tumors, however, one study found that 38.5% of postoperative green turtle patients regrew tumors at removal sites within 36 days of surgery (40). In our study, after treatment with the autovaccine, followed-up with surgical removal of the fibropapilloma, the FP did not present a recurrence. A full recovery was observed after 6 months follow up period and the liberation of the turtle was recommended. Further investigation is needed in order to confirm our results in other individuals.

## Data availability statement

The datasets presented in this study can be found in online repositories. The names of the repository/repositories and accession number(s) can be found at: https://www.ncbi.nlm.nih.gov/genbank/, OR259410.

## Ethics statement

The animal study was approved by Ethics Committee/Universidad del Magdalena. The study was conducted in accordance with the local legislation and institutional requirements.

## Author contributions

LC: Conceptualization, Data curation, Formal analysis, Funding acquisition, Investigation, Methodology, Project administration, Resources, Supervision, Validation, Writing – original draft, Writing – review & editing. VV: Formal analysis, Investigation, Methodology, Writing – review & editing. ÁO: Formal analysis, Investigation, Methodology, Writing – review & editing. EZ: Investigation, Methodology, Writing – review & editing. AD: Investigation, Methodology, Writing – review & editing. GN: Investigation, Methodology, Writing – review & editing. BO: Investigation, Methodology, Writing – review & editing. AC: Investigation, Methodology, Writing – review & editing. JP: Investigation, Methodology, Writing – review & editing. AO: Investigation, Methodology, Writing – review & editing. NV: Conceptualization, Formal analysis, Funding acquisition, Investigation, Methodology, Project administration, Resources, Writing – original draft, Writing – review & editing.

## Footnotes

^1^ https://www.iucnredlist.org/

^2^ https://blast.ncbi.nlm.nih.gov/
