# Supplementary figures and images for: Case report: Diagnosis and autogenous vaccine treatment of herpesvirus in a green turtle (Chelonia mydas) in Santa Marta, Colombia
Source: Front Vet Sci. 2024 Jan 31;11:1258209. doi: 10.3389/fvets.2024.1258209 (PMC10880012; doi:10.3389/fvets.2024.1258209)

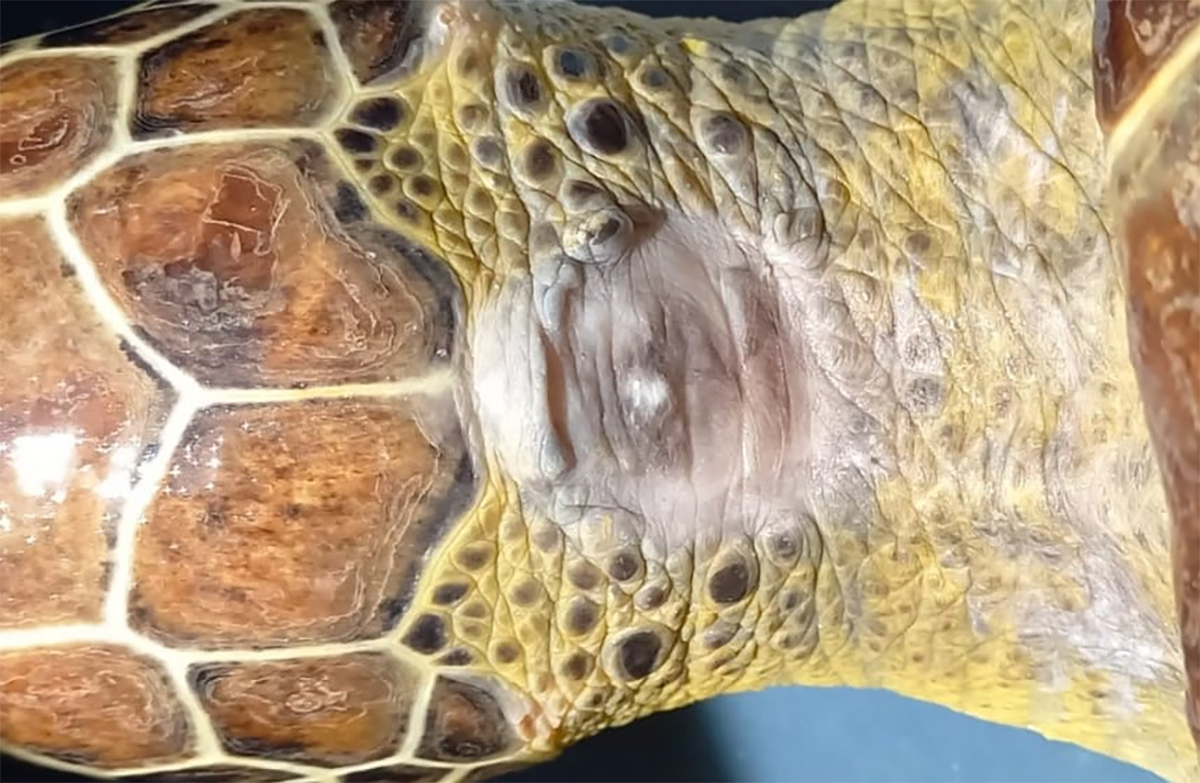

Supplement: Supplementary file 1 [file Image_1.JPEG]
