# Supplementary material for: Case report: Diagnosis and autogenous vaccine treatment of herpesvirus in a green turtle (Chelonia mydas) in Santa Marta, Colombia
Source: Front Vet Sci. 2024 Jan 31;11:1258209. doi: 10.3389/fvets.2024.1258209 (PMC10880012; doi:10.3389/fvets.2024.1258209)
Supplement: Supplementary file 2 [file Table_1.docx]

Supplementary Table 1. Green turtle *Chelonia mydas* vaccination scheme with autovaccine 1, cycle 1 and cycle 2 (A), Green turtle *Chelonia mydas* vaccination scheme with autovaccine 2 (B).

A.

| **Date of treatment – Cycle 1** | **Doses** | **Date of treatment – Cycle 2** | **Doses** |
| --- | --- | --- | --- |
| 29-Mar-21 | 1 ml | 1-May-21 | 1 ml |
| 30-Mar-21 | 2 ml | 2-May-21 | 2 ml |
| 31-Mar-21 | 3 ml | 3-May-21 | 3 ml |
| 1-Apr-21 | 4 ml | 4-May-21 | 4 ml |
| 2-Apr-21 | 3 ml | 5-May-21 | 3 ml |
| 3-Apr-21 | 2 ml | 6-May-21 | 2 ml |
| 4-Apr-21 | 1 ml | 7-May-21 | 1 ml |

B.

| **Date of treatment** | **Doses** |
| --- | --- |
| 1-Sep-22 | 0.5 ml |
| 5-Sep-22 | 0.5 ml |
| 7-Sep-22 | 0.5 ml |
| 9-Sep-22 | 0.5 ml |
| 12-Sep-22 | 0.5 ml |
| 14-Sep-22 | 0.5 ml |
| 16-Sep-22 | 0.5 ml |
| 19-Sep-22 | 1 ml |
| 23-Sep-22 | 1 ml |
| 27-Sep-22 | 1 ml |
| 4-Sep-22 | 1 ml |
